# Correction: Synthesis of nanostructured catalysts by surfactant-templating of large-pore zeolites

**DOI:** 10.1039/c9na90049g

**Published:** 2019-09-18

**Authors:** Aqeel Al-Ani, Josiah J. C. Haslam, Natalie E. Mordvinova, Oleg I. Lebedev, Aurélie Vicente, Christian Fernandez, Vladimir Zholobenko

**Affiliations:** School of Chemical and Physical Sciences, Keele University Keele Staffordshire ST5 5BG UK a.a.t.al-ani@keele.ac.uk; Oil Marketing Company (SOMO) Baghdad Iraq; Laboratoire CRISMAT, ENSICAEN, UMR CNRS 6508 6 Boulevard du Maréchal Juin 14050 Caen Cedex 04 France; Normandie Univ, ENSICAEN, UNICAEN, CNRS, Laboratoire Catalyse et Spectrochimie 14000 Caen France

## Abstract

Correction for ‘Synthesis of nanostructured catalysts by surfactant-templating of large-pore zeolites’ by Aqeel Al-Ani *et al.*, *Nanoscale Adv.*, 2019, **1**, 2029–2039.

The following amendments have been suggested:

## Introduction

The surfactant-templated mesostructuring approach, introduced in ref. 21 and 25, allows for a more precise control of the intracrystalline mesoporosity introduced while retaining the main properties of the zeolites, including their microporosity, catalytic activity, and hydrothermal stability. For these reasons, surfactant-templated zeolites have been successfully commercialised.^22,36^

## Experimental section

The preparation of mesostructured faujasites followed the general procedure introduced in ref. 25.

## Results and discussion

In agreement with previous reports,^18,25,44^ our results demonstrate that TIPB conversion increases for all zeolites following their mesostructuring treatment.

## Acknowledgments

The authors would like to thank Professor Javier Garcia-Martinez and Dr Eric Li for the useful information provided regarding the synthesis of surfactant-templated zeolites.

## References

The reference numbers cited here correspond to those in the original paper.

The Royal Society of Chemistry apologises for these errors and any consequent inconvenience to authors and readers.

## Supplementary Material

